# The Pan-American Medical Congress

**Published:** 1892-04

**Authors:** 


### THE PAN-AMERICAN MEDICAL CONGRESS.

We publish, on another page, a circular of information from the Secretary-General, Dr. Charles A. L. Reed, though it is signed in his capacity as Chairman of the Committee of Organization. This is simply because it contains the preliminary features pertaining to the organization of the Congress that were inaugurated under the Committee before he was elected Secretary-General. This is probably the last circular that will be signed in this way, as in the future all his duties will be performed under his authority as Secretary-General. We trust that all interested in this Congress will read carefully the general and special regulations, that they may familiarize themselves with their details, and especially is it considered important that those who intend to become members shall forward their registration fee to the Treasurer, Dr. A. M. Owen, Evansville, Ind., without delay.

The incorporation of the Congress under the laws of Ohio was a wise act on the part of the officers, because it removes it from the shadow or control of any organization, clique, or association. It is now a Congress pure and simple, acting for itself, with its own autonomy, under the authority of law, and can both receive and bestow contributions of money without danger of being charged with illegality by such acts. The Congress has made such rapid progress towards organization in other countries, that it will be very easy to organize all the work pertaining to the United States as soon as the committee shall have made its next report to the American Medical Association, at which time its relations to that body will naturally cease. We bespeak for it the encouragement that it deserves from every American physician, as well as every good citizen.

